# HLA-B*58:01 screening in Asia-Pacific is an ethical imperative – not just a cost question

**DOI:** 10.7189/jogh.15.03037

**Published:** 2025-08-22

**Authors:** Wei Leik Ng, Emily Tsui Yee Tse, Prawira Oka, Hooi Min Lim, Sky Wei Chee Koh, Rizawati Ramli, Hung Chiun Lau, Benjamin Chih Chiang Lam, Laurie J Goldsmith, Adina Abdullah, Lay Hoon Goh

**Affiliations:** 1 Department of Primary Care Medicine, Faculty of Medicine, Universiti Malaya, Kuala Lumpur, Malaysia; 2 Department of Family Medicine and Primary Care, School of Clinical Medicine, Li Ka Shing Faculty of Medicine, The University of Hong Kong, Hong Kong, China; 3 SingHealth Polyclinics, Singapore, Singapore; 4 SingHealth-Duke NUS Family Medicine Academic Clinical Program, Singapore, Singapore; 5 National University Polyclinics, National University Health System, Singapore, Singapore; 6 Division of Family Medicine, Department of Medicine, Yong Loo Lin School of Medicine, National University of Singapore, Singapore, Singapore; 7 Department of Family Medicine, Faculty of Medicine and Health Sciences, Universiti Putra Malaysia, Selangor, Malaysia; 8 Family and Community Medicine Section, Department of General Medicine, Khoo Teck Puat Hospital, Singapore, Singapore

## Abstract

Allopurinol remains the first-line treatment for gout; however, it carries a risk of severe cutaneous adverse reactions (SCARs), particularly among carriers of the HLA-B*58:01 allele. In the Asia-Pacific region, screening practices vary widely due to infrastructure gaps, cost concerns, and differing health policies. For example, while cost-effectiveness analyses have influenced screening decisions in some countries, they often overlook long-term health system burdens and ethical imperatives. Therefore, screenings should not be withheld solely on economic grounds, especially when they can prevent life-threatening outcomes. For that reason, a targeted, risk-based approach that integrates genetic and clinical factors could offer a practical and equitable path forward, improving pharmacogenomic literacy, expanding access to testing, developing regional data-sharing platforms, and involving patients in co-designing screening strategies. Rapid point-of-care testing and integration of screening process with existing care pathway may further support this implementation. However, a coordinated regional effort is needed to ensure safer prescribing and equitable access to pharmacogenomic screening across diverse healthcare settings.

## BURDEN OF ALLOPURINOL-INDUCED SEVERE CUTANEOUS ADVERSE REACTION

Gout affects 1–6.8% of the global population and is becoming more prevalent due to metabolic syndrome and dietary changes [1]. Allopurinol remains the first-line treatment, but it can cause severe cutaneous adverse reactions (SCAR), including Stevens-Johnson syndrome (SJS), toxic epidermal necrolysis (TEN), and drug reaction with eosinophilia and systemic symptoms (DRESS). Allopurinol-induced SCAR poses a significant burden in the Asia-Pacific region [2], with Malaysia and Singapore reporting annual incidence rates of 2.5–2.6 per 1000 users – relatively high for a rare, but serious reaction [3,4]. Though uncommon, SCAR can be fatal, with mortality rates of 2–10% for SJS and DRESS, and up to 30% for TEN [5,6].

## GENETIC SCREENING AND THE CASE FOR TARGETED SCREENING

Allopurinol-induced SCAR is strongly associated with the HLA-B*58:01 allele, with studies from several Asia-Pacific countries reporting odds ratios up to 393 [7–11]. The allele is most prevalent among individuals of Chinese ethnicity (12–22%), with consistent rates across Malaysia, Singapore, and Hong Kong [12–14]. Other populations, including Thai, Korean, and Javanese Indonesians, also carry the allele (typically 5–12%) [15–17]. The prevalence of the HLA-B*58:01 allele corresponds with the higher incidence of allopurinol-induced SCAR observed in countries such as Malaysia and Singapore [3,4]. Chinese patients are disproportionately affected in both settings; however, Malays also exhibit a significant risk and should be considered in screening strategies. In contrast, SCAR cases among Indian populations are less frequently reported, which is consistent with the lower prevalence of the allele in this group. There is currently no evidence linking HLA-B*58:01 to SCAR among the Malay ethnic group in Indonesia, despite such associations being observed among Malays in Malaysia [18]. Data from certain countries such as India and the Philippines is limited. This highlights the ethnic diversity across the Asia-Pacific region and the incomplete evidence base for all populations. These gaps underscore the need for more population-specific research to establish genetic risk and support a targeted screening approach adapted to local epidemiology. Importantly, some HLA-B*58:01 carriers tolerate allopurinol without adverse effects [14], while other factors, such as age, sex, renal impairment and initial dosage, also contribute to elevated SCAR risk [9,19]. These complexities highlight the need for a multifactorial risk assessment that integrates both genetic and clinical factors.

## LOCAL GUIDELINES AND THE INFLUENCE OF COST-EFFECTIVENESS

International guidelines generally support HLA-B*58:01 screening before starting allopurinol, but stop short of recommending universal testing. The American College of Rheumatology advises screening for high-risk groups, such as Southeast Asians and African Americans [20], while the Clinical Pharmacogenetics Implementation Consortium and the Dutch Pharmacogenetics Working Group recommend targeted screening without specifying populations [21,22].

In the Asia-Pacific region, national approaches vary. Singapore’s guidelines cite low predictive value and the role of non-genetic risk factors, suggesting screening only among high-risk individuals, such as the elderly or those with kidney disease, while considering the screening cost [23]. Malaysia acknowledges the genetic risk but does not support routine screening due to cost concerns and lacks specific guidance for high-risk groups [24]. In contrast, Hong Kong’s 2023 guideline recommends screening for certain Asian and African American patients, especially those above 60 years of age or with renal impairment [25].

Most guidelines emphasise ethnicity and renal function, but often overlook individual drug tolerance, multiethnic variation, equity concerns, and practical implementation challenges. Cost-effectiveness remains a central consideration, particularly in Malaysia and Singapore, where earlier studies concluded that HLA-B*58:01 screening was not cost-effective, even among patients with renal impairment [26,27]. These recommendations often focussed on the limited predictive value of the test, using this as a rationale against broader screening. However, past evaluations omit the long-term costs associated with severe SCAR outcomes, including dialysis and chronic care. By excluding these downstream impacts, current models may underestimate the true value of targeted screening, especially for high-risk groups.

## SCREENING FOR HLA-B*58:01 IN THE ASIA PACIFIC REGION: VARIATION IN PRACTICE

Across the Asia-Pacific region, HLA-B*58:01 screening is not routinely performed before initiating allopurinol, barring a few exceptions. For instance, Korea and Thailand support universal HLA-B*58:01 testing through public insurance. Specifically, Thailand’s primary care is mainly hospital-based, thus screening may be conducted onsite or referred to larger hospitals, depending on local infrastructure.

Hong Kong has taken more systematic steps by implementing universal screening in its public healthcare system since March 2023, following a sentinel SCAR-related death. Its electronic prescribing system prompts clinicians to order HLA-B*58:01 testing before initiating allopurinol. In Singapore, routine screening is not performed in primary care but may be offered in tertiary centres for high-risk patients. Meanwhile, in Malaysia, screening is uncommon in both public and private practices, often requiring special arrangements and incurring additional costs.

Despite strong genetic associations with SCAR, adoption of HLA-B*58:01 screening remains uneven across the region due to multiple interrelated challenges. Implementation is often inconsistent, shaped by a lack of physician awareness, resource availability, and local workflows. Primary care infrastructure frequently lacks capacity for genetic testing, with services concentrated in tertiary centres. Physicians face uncertainty about whom to screen and how to manage positive results, reflecting gaps in clinical protocols and pharmacogenomic literacy. Patient awareness is low, and reluctance to pay out-of-pocket further limits uptake in countries where screening is not supported by national health insurance or public funding, such as Malaysia and Singapore. Even when screening is available, access to safer alternatives like febuxostat is constrained by cost and limited availability in public clinics. While febuxostat demonstrates better efficacy and safety compared to other second-line urate-lowering agents, it carries an US Food and Drug Administration (FDA) black-box warning for increased cardiovascular events and death [20]. Although Mackenzie and colleagues suggest no greater cardiovascular risk with febuxostat, neither the European Medicines Agency nor the FDA have revised their warnings, continuing to advise against prescribing febuxostat to patients with a history of cardiovascular events [28]. These regulatory barriers, combined with fragmented funding, contribute to sporadic screening implementation.

## BALANCING ETHICS, EQUITY, AND PRACTICALITY IN SCREENING IMPLEMENTATION

From an ethical standpoint, it is neither justifiable nor equitable to forgo HLA-B*58:01 screening solely due to cost, especially when existing cost-effectiveness analyses may underestimate long-term health system burdens and when patients are expected to pay out-of-pocket. Financial barriers disproportionately affect vulnerable populations and undermine equitable access to safe treatment. However, ethical action must also be context sensitive. In resource-limited settings or among populations with demonstrated tolerance to allopurinol despite HLA-B*58:01 positivity, universal screening may not be the most practical or just approach. Evidence shows that individuals carrying this allele tolerate allopurinol without adverse effects [14], and that blanket exclusion could deny access to an affordable and effective therapy, particularly where alternatives like febuxostat are costly or carry additional risks. Therefore, a comprehensive economic evaluation that incorporates long-term health system costs is needed to assess the value of this screening approach, while cost-effectiveness may also increase as generic febuxostat prices decline in the future.

Moreover, a risk-based approach that considers non-genetic factors such as age, renal function, ethnicity, and genetic markers could optimise screening safety without overburdening the system. Developing a robust risk prediction model that incorporates both genetic and non-genetic factors would be valuable in this sense. However, regional pharmacovigilance data remain fragmented and incomplete; although existing platforms share genetic data such as HLA allele prevalence, broader pharmacovigilance reporting is not yet harmonised. Efforts are ongoing to foster regional collaboration in pharmacogenomics, but have yet to mature into sustained data-sharing systems. Establishing an Asia-Pacific regional registry that incorporates pharmacovigilance data and genetic markers would improve data quality and support the development of more accurate and cost-effective screening models. In Malaysia and Singapore, screening strategies should also consider clinical and demographic factors beyond ethnicity and kidney disease to enhance relevance and cost-effectiveness.

Importantly, patients should be involved in co-designing screening strategies to ensure alignment with their values and experiences. Collaboration with gout advocacy groups could lead to more inclusive policies, such as subsidised genetic testing, public education on SCAR, and routine use of pharmacogenomic safety measures. This involvement is especially vital in low- and middle-income countries (LMICs), where access and awareness are limited.

Tiered screening for HLA-B*58:01 may be feasible in LMICs, and rural areas with limited access to genetic testing facilities. These settings could consider the deployment of point-of-care (POC) tests, with positive results referred to advanced centres for confirmatory testing. To our knowledge, no POC kits have received regulatory approval, though several are in development and may enable safer prescribing once validated. Integration into routine care workflows is also essential. Hong Kong’s electronic medical record alert system, which prompts HLA-B*58:01 testing before allopurinol initiation, serves as a model for embedding pharmacogenomic safety into clinical decision-making.

Finally, we call on the US FDA to re-evaluate its boxed warning on febuxostat to address the conflicting findings in the Cardiovascular Safety of Febuxostat and Allopurinol in Patients with Gout and Cardiovascular Morbidities *vs.* Febuxostat *vs.* Allopurinol Streamlined Trial [28,29]. Given the limited Asian representation in earlier trials, a population-specific review is warranted to ensure equitable access to alternative urate-lowering therapies for high-risk groups.

## CONCLUSIONS

Addressing the burden of allopurinol-induced SCAR in the Asia-Pacific region requires a multifaceted strategy that incorporates genetic screening, assessment of clinical risk factors, and ensures equitable access to safer therapeutic alternatives. Regional collaboration, improved pharmacovigilance, and patient-centred approaches, including advocacy and co-design, could help shape inclusive, cost-effective screening policies.
